# Population-based model selection for an accurate estimation of time-integrated activity using non-linear mixed-effects modelling

**DOI:** 10.1016/j.zemedi.2023.01.007

**Published:** 2023-02-20

**Authors:** Deni Hardiansyah, Ade Riana, Matthias Eiber, Ambros J. Beer, Gerhard Glatting

**Affiliations:** a Medical Physics and Biophysics, Physics Department, Faculty of Mathematics and Natural Sciences, Universitas Indonesia, Depok, Indonesia; b Research Collaboration Center for Theranostic Radiopharmaceuticals, BRIN, Bandung, Indonesia; c Department of Nuclear Medicine, Klinikum rechts der Isar, Technische Universität München, München, Germany; d Department of Nuclear Medicine, Ulm University, Ulm, Germany; e Medical Radiation Physics, Department of Nuclear Medicine, Ulm University, Ulm, Germany

**Keywords:** Akaike weight, Model selection, NLME, PSMA

## Abstract

**Purpose:**

Personalized treatment planning in Molecular Radiotherapy (MRT) with accurately determining the absorbed dose is highly desirable. The absorbed dose is calculated based on the Time-Integrated Activity (TIA) and the dose conversion factor. A crucial unresolved issue in MRT dosimetry is which fit function to use for the TIA calculation. A data-driven population-based fitting function selection could help solve this problem. Therefore, this project aims to develop and evaluate a method for accurately determining TIAs in MRT, which performs a Population-Based Model Selection within the framework of the Non-Linear Mixed-Effects (NLME-PBMS) model.

**Methods:**

Biokinetic data of a radioligand for the Prostate-Specific Membrane Antigen (PSMA) for cancer treatment were used. Eleven fit functions were derived from various parameterisations of mono-, bi-, and tri-exponential functions. The functions' fixed and random effects parameters were fitted (in the NLME framework) to the biokinetic data of all patients. The goodness of fit was assumed acceptable based on the visual inspection of the fitted curves and the coefficients of variation of the fitted fixed effects. The Akaike weight, the probability that the model is the best among the whole set of considered models, was used to select the fit function most supported by the data from the set of functions with acceptable goodness of fit. NLME-PBMS Model Averaging (MA) was performed with all functions having acceptable goodness of fit. The Root-Mean-Square Error (RMSE) of the calculated TIAs from individual-based model selection (IBMS), a shared-parameter population-based model selection (SP-PBMS) reported in the literature, and the functions from NLME-PBMS method to the TIAs from MA were calculated and analysed. The NLME-PBMS (MA) model was used as the reference as this model considers all relevant functions with corresponding Akaike weights.

**Results:**

The function $f_{3a}=A_{1}{\;e}^{-\left(\lambda_{1}+\lambda_{\mathrm{phys}}\right)t}+A_{2}{\;e}^{-\left(\lambda_{\mathrm{phys}}\right)t}$ was selected as the function most supported by the data with an Akaike weight of (54 ± 11) %. Visual inspection of the fitted graphs and the RMSE values show that the NLME model selection method has a relatively better or equivalent performance than the IBMS or SP-PBMS methods. The RMSEs of the IBMS, SP-PBMS, and NLME-PBMS ($f_{3a}$) methods are 7.4%, 8.8%, and 2.4%, respectively.

**Conclusion:**

A procedure including fitting function selection in a population-based method was developed to determine the best fit function for calculating TIAs in MRT for a given radiopharmaceutical, organ and set of biokinetic data. The technique combines standard practice approaches in pharmacokinetics, i.e. an Akaike-weight-based model selection and the NLME model framework.

## 1 Introduction

The estimation of individual absorbed doses in molecular radiotherapy (MRT) is generally helpful for therapy optimisation [1], [2], [3], [4]. For example, the EC Directive 2013/59/Euratom states that absorbed doses (requiring time-integrated activity TIAs as input) in nuclear medicine therapies shall be planned individually [5]. A critical unresolved issue in MRT dosimetry is the question of which fit function to use for the calculation of the TIA, as the obtained TIA value highly depends on the chosen fit function [6], [7]. Therefore, determining the “optimal” fit function is important for accurately calculating TIAs and, subsequently, the absorbed dose. The “optimal” fit function can be selected from a set of suitable models using a model selection procedure [8]. Relevant criteria for the best fit function are that the goodness of fit is satisfactory and that this function is most supported by the data [7]. The latter requires model (or function) selection that could be done using the Akaike Information Criterion (AIC) method [9], [10].

The measurement of biokinetic data is often done at only a few time points in the clinic. The selection of a fit function for an individual patient, i.e. individual-based model selection (IBMS), based on a low number of data, allows only the investigation of fit functions having few parameters, which, therefore, may not correctly reflect the kinetics of the radiopharmaceutical [6]. In addition, model selection has a high uncertainty for a low ratio of the number of data to the number of adjustable parameters. Therefore, population-based model selection is of interest as it can increase the ratio between the number of input data and the number of parameters, which could lead to lower uncertainty in model selection. Recently, we developed a population-based model selection method with a shared function parameter (SP-PBMS) that could lead to a better performance than the IBMS method [6]. The fitting was performed population-wise, including functions with a parameter shared between patients.

Another population-based approach used for TIAs calculation is the non-linear mixed-effects (NLME) modelling. Devasia et al. recently used an NLME model to determine kidney TIAs in PRRT with ^177^Lu-DOTATATE using a bi-exponential function and biokinetic data measured with SPECT/CT. The NLME model provides valuable information across patients (inter-individual variability) and within patients (intra-individual variability) that could be used to optimise the individual estimation of TIAs [11]. It has been shown that implementing NLME modelling could lead to an acceptable accuracy of calculated TIAs [11]. Although the NLME model has been shown to perform well [11], [12], the bi-exponential function used in the NLME method might not be applicable to biokinetic data of different radiopharmaceuticals, organs, or patient biokinetic data. Therefore, using the best fit function within the NLME model approach may improve the NLME method.

In this work, we present a general method based on population information to improve the calculation of TIAs. It is demonstrated in an example of ^177^Lu-PSMA therapy using the NLME model and population-based model selection (NLME-PBMS) method. In this NLME-PBMS method, a set of mathematical models (or functions) is defined, an NLME model fit is performed, and the best fit function is selected using the Akaike weight method [13], [14]. In addition, the performance of the NLME-PBMS method is compared to the IBMS and the SP-PBMS methods reported in the literature [6]. In the clinic, the NLME-PBMS method can be used to define the best fit function for a given radiopharmaceutical, organ and population kinetic data set that can be used to fit a “new” patient with the known inter- and intra-individual variability.

## 2 Materials and methods

### 2.1 Biokinetic data

Biokinetic data (the time-activity data) of [^177^Lu]Lu-PSMA-I&T RLT in kidneys were collected from thirteen patients with metastatic castration-resistant prostate cancer [6], [15]. All patients underwent [^177^Lu]Lu-PSMA-I&T radioligand therapy (RLT) and had post-therapeutic planar whole-body scintigraphies at (1.1 ± 0.7) h, (20.7 ± 2.3) h, and (163.8 ± 2.0) h p.i. One patient had one additional time point at 66.1 h p.i. Three patients had two extra time points at (45.9 ± 1.6) h and (68.7 ± 1.7) h p.i. The activity was quantified from the images by drawing the regions of interest using the geometric mean of anterior and posterior counts with background corrections [6], [15]. There was no scatter correction performed. Calibration factors were calculated based on the whole-body region of interest in the first scan. The institutional review board approved this retrospective analysis (115/18S), and the requirement for informed consent was waived.

### 2.2 Set of sums of exponential functions

As suggested by Burnham et al., only well-suited functions should be included in the investigated function set [8]. Therefore, sums of exponential functions (one, two and three terms) [6], [7] with increasing complexity were used and analysed to describe the physical and biological processes of the radiopharmaceutical’s pharmacokinetics (Eqs. (1), (2), (3), (4), (5), (6), (7), (8), (9), (10), (11)):

(1) $$f_{2a}\left(t\right)=A_{1}e^{-\left(\lambda_{1}+\lambda_{\mathrm{phys}}\right)t}$$

(2) $$f_{3a}\left(t\right)=A_{1}e^{-\left(\lambda_{1}+\lambda_{\mathrm{phys}}\right)t}+A_{2}e^{-\left(\lambda_{\mathrm{phys}}\right)t}$$

(3) $$f_{3b}\left(t\right)=A_{1}\alpha e^{-\left(\lambda_{1}+\lambda_{\mathrm{phys}}\right)t}+A_{1}\left(1-\alpha \right)e^{-\left(\lambda_{\mathrm{phys}}\right)t}$$

(4) $$f_{3c}\left(t\right)=A_{1}\left(1-\alpha \right)e^{-\left(\lambda_{1}+\lambda_{\mathrm{phys}}\right)t}+A_{1}\alpha e^{-\left(\lambda_{\mathrm{phys}}\right)t}$$

(5) $$f_{4a}\left(t\right)=A_{1}e^{-\left(\lambda_{1}+\lambda_{\mathrm{phys}}\right)t}+A_{2}e^{-\left(\lambda_{2}+\lambda_{\mathrm{phys}}\right)t}$$

(6) $$f_{4b}\left(t\right)=A_{1}{\alpha e}^{-\left(\lambda_{1}+\lambda_{\mathrm{phys}}\right)t}+A_{1}\left(1-\alpha \right)e^{-\left(\lambda_{2}+\lambda_{\mathrm{phys}}\right)t}$$

(7) $$f_{5a}\left(t\right)=A_{1}e^{-\left(\lambda_{1}+\lambda_{\mathrm{phys}}\right)t}+A_{2}e^{-\left(\lambda_{2}+\lambda_{\mathrm{phys}}\right)t}-A_{3}e^{-\left(\lambda_{\mathrm{phys}}\right)t}$$

(8) $$f_{5b}\left(t\right)=A_{1}e^{-\left(\lambda_{1}+\lambda_{\mathrm{phys}}\right)t}+A_{2}e^{-\left(\lambda_{2}+\lambda_{\mathrm{phys}}\right)t}-\left(A_{2}+A_{1}\right)e^{-\left(\lambda_{3}+\lambda_{\mathrm{phys}}\right)t}$$

(9) $$f_{5c}\left(t\right)=A_{1}e^{-\left(\lambda_{1}+\lambda_{\mathrm{phys}}\right)t}+A_{2}e^{-\left(\lambda_{2}+\lambda_{\mathrm{phys}}\right)t}+A_{3}e^{-\left(\lambda_{\mathrm{phys}}\right)t}$$

(10) $$f_{6a}\left(t\right)=A_{1}e^{-\left(\lambda_{1}+\lambda_{\mathrm{phys}}\right)t}+A_{2}e^{-\left(\lambda_{2}+\lambda_{\mathrm{phys}}\right)t}-A_{3}e^{-\left(\lambda_{3}+\lambda_{\mathrm{phys}}\right)t}$$

(11) $$f_{6b}\left(t\right)=A_{1}e^{-\left(\lambda_{1}+\lambda_{\mathrm{phys}}\right)t}+A_{2}e^{-\left(\lambda_{2}+\lambda_{\mathrm{phys}}\right)t}+A_{3}e^{-\left(\lambda_{3}+\lambda_{\mathrm{phys}}\right)t}$$

where $f_{i}$ is a fit function with i estimated parameters, the $A_{j}$ are pre-factors with values ≥0, $\lambda_{\mathrm{phys}}$ is the physical decay constant of ^177^Lu ($\lambda_{\mathrm{phys}}=\ln\left(2\right)/T_{1/2}$, with the half-life $T_{1/2}$=6.6443 d [16]), $\lambda_{j}$ are the biological decay parameters of the radiopharmaceutical. All biological decay constants $\lambda_{j}$ were estimated with a value ≥0 as described in the literature [17], [18].

### 2.3 Non-linear mixed-effects model

The NLME model was used to estimate the parameters of the SOE functions in Eqs. (1), (2), (3), (4), (5), (6), (7), (8), (9), (10), (11), i.e. $A_{j}$ and $\lambda_{j}$. The estimated parameters in the NLME model were defined as a combination of the fixed and random effects. Fixed effects are the mean values of the estimated parameters in the population, while random effects show the variability of the estimated parameters [19]:

(12) $$P_{i}=TVP_{i}\times exp\left({\mathrm{ETA}}_{i}\right)$$

(13) $${\mathrm{ETA}}_{i}=N\left(0,{\sigma_{i}}^{2}\right)$$

Here $P_{i}$ is the estimated parameter i, $\mathrm{TV}P_{i}$ is the fixed effect of the estimated parameter i in the population, and ${\mathrm{ETA}}_{i}$ is a random number following a Gaussian distribution with mean zero and variance ${\sigma_{i}}^{2}$ (random effect) [11], [12], [20].

### 2.4 Study workflow

The parameters of Eqs. (1), (2), (3), (4), (5), (6), (7), (8), (9), (10), (11) were fitted to the biokinetic data of [^177^Lu]Lu-PSMA-I&T RLT in kidneys using the NLME method. All fittings were performed in MATLAB software vR2020a. The exponential error model was used for the NLME model fittings [12]. The following computational settings were used for the IBMS and SP-PBMS methods as suggested in the literature [6]: Rosenbrock Algorithm, objective function convergence criterion 10^−4^, and absolute-based variance model with a fractional standard deviation of 0.15. Starting values of the parameters were chosen based on a trial-and-error approach. The goodness of fit for each SOE function was checked by visual inspection of the fitted graphs and the coefficient of variation CV of the fitted fixed-effects parameters (Table 1 in ref. [7], goodness of fit acceptable for CV < 0.5). The best fit function selection was performed by calculating the corrected Akaike Information Criterion AICc, which is the AIC corrected for the case of N/K < 40 (number of data N and number of parameters K) [8], [9]. The corresponding Akaike weights [7], [8], [9] were calculated as follows:

(14) $$\mathrm{AICc}=-2\ln\left(P\right)+2K+\frac{2K(K+1)}{N-K-1}$$

(15) $$\Delta_{i}={\mathrm{AICc}}_{i}-{\mathrm{AICc}}_{\min}$$

(16) $$w_{{\mathrm{AICc}}_{i}}=e^{-\frac{\Delta_{i}}{2}}/\sum_{i=i}^{F}e^{-\frac{\Delta_{i}}{2}}$$

where P is the objective function minimized for the fitting, ${\mathrm{AICc}}_{\min}$ is the lowest AICc value of all fitted functions, $\Delta_{i}$ is the difference of the ${\mathrm{AICc}}_{i}$ of function i and the ${\mathrm{AICc}}_{\min}$, F is the total number of functions that passed the goodness-of-fit test and $w_{{\mathrm{AICc}}_{i}}$ is the Akaike weight of function i. The Akaike weights indicate the probability that the model is the best among the whole set of considered models [8], [9]. To analyse the stability of the NLME-PBMS method through model selection for different set of data, the Jackknife method was used as suggested in the literature [9], [21]. Here, the leave-one-out method was applied thirteen times with only twelve patients included for the calculation of the Akaike weights.

The best function in the IBMS method for the same biokinetic data was function $f_{2a}$ (Eq. (1)) [6]. In the SP-PBMS method, the best function describing the biokinetic data of [^177^Lu]Lu-PSMA-I&T was function $\overset{¯}{f_{3b}}$ (i.e. $f_{3b}$, Eq. (3) with α= 0.9632) [6]. Therefore, we used functions $f_{2a}$ and $\overset{¯}{f_{3b}}\;$ to describe the biokinetics of [^177^Lu]Lu-PSMA-I&T in kidneys to represent IBMS and SP-PBMS methods, respectively. The TIAs were calculated using the functions from IBMS, SP-PBMS and NLME-PBMS methods with integration from *t* = 0 min to t = 100 000 min. The standard deviations of the TIAs from the IBMS and SP-PBMS method were calculated based on the bootstrap method using the frequency-based sampling for the parameters as suggested by Saltelli et al. [22]:

(17) $$X_{i}\left(s\right)=G_{i}\left(\sin\left(\omega_{i}s\right)\right)=\frac{1}{2}+\frac{1}{\pi }arcsin\left(\sin\left(\omega_{i}s+\varphi_{i}\right)\right)$$

where s is a modified scalar variable varying over the range -π/2 < s<π/2, and $\omega_{i}$ is the frequency value used for parameter $X_{i}$ and $\varphi_{i}$is a random phase-shift chosen uniformly between 0 and 2π. Parameters of the SOE function $X_{i}$ were sampled from the variability obtained from the IBMS and SP-PBMS methods. The frequencies show a specific number related to the sinusoidal functions assigned to each parameter [23]. The vector of the input parameters of interest $X_{i}$ was sampled from the log-normal distribution (Eqs. (18), (19)). The sampling of the IBMS and SP-PBMS fitted parameters were generated using Eq. (17). The mean and SD of the parameters in the log-normal distribution were calculated based on the following equations [23]:

(18) $${\mathrm{mean}}_{\log-normal}=2\times ln\left({\mathrm{mean}}_{\mathrm{normal}}\right)-0.5\times ln\left({\mathrm{mean}}_{\mathrm{normal}}^{2}+{\mathrm{SD}}_{\mathrm{normal}}^{2}\right)$$

(19) $${\mathrm{SD}}_{\log-normal}=\sqrt{\ln\left({\mathrm{mean}}_{\mathrm{normal}}^{2}+{\mathrm{SD}}_{\mathrm{normal}}^{2}\right)-2\times ln\left({\mathrm{mean}}_{\mathrm{normal}}\right)}$$

where ${\mathrm{mean}}_{\log-normal}$ is the mean of the parameters in log-normal distribution, ${\mathrm{SD}}_{\log-normal}$ is the SD of the parameters in log-normal distribution, ${\mathrm{mean}}_{\mathrm{normal}}$ is the mean of the parameters in normal distribution, and ${\mathrm{SD}}_{\mathrm{normal}}$ is the SD of the parameters in normal distribution. The frequency $\omega_{i}$ and the number of model evaluations in Eq. (17) were fixed to 1028 and 8193 [23], respectively. The standard deviations of individual TIAs from the NLME-PBMS method was not calculated as the NLME model fitting method only reports the intra-individual variability from the populations [12], [19].

The TIA was also calculated for the model averaging (MA) [8], [10] from the NLME-PBMS method according to the following equation:

(20) $${\mathrm{TIA}}_{\mathrm{MA}}=\sum_{i}w_{{\mathrm{AICc}}_{i}}\times {\mathrm{TIA}}_{i}$$

where ${\mathrm{TIA}}_{\mathrm{MA}}$ is the TIA of MA, $w_{{\mathrm{AICc}}_{i}}$ is the Akaike weight of the SOE function i calculated using Eq. (16), and ${\mathrm{TIA}}_{i}$ is the TIA of the SOE function i in the NLME-PBMS method. Relative deviations (RDs) and root-mean-square errors (RMSEs) were used to analyse the accuracy of the calculated TIAs using the IBMS, SP-PBMS, and NLME-PBMS methods (per single fit function) with the TIAs obtained from the NLME-PBMS (MA) method as the reference. The total number of fitted parameters leads to the superiority of the NLME-PBMS method over the IBMS and SP-PBMS methods. For the same data, the total number of parameters in the NLME-PBMS, IBMS and the SP-PBMS method are K = 7 (Table 1), K = 26 (Function $f_{2b}$in [6]), and K = 27 (Table 1 in [6]) respectively. Based on Eq. (14), with a conservative assumption of equal −2 ln (P) values for all fits and N = 46 data points, the values of the term $\left(2K+\frac{2K\left(K+1\right)}{N-K-1}\right)$ for the NLME-PBMS, IBMS and SP-PBMS methods are 17, 126, and 138, respectively. These large differences in the AICc leads to Akaike weights (Eqs. (15), (16)) of the best function from the NLME-PBMS, IBMS and SP-PBMS models of about 100%, 2.2 × 10^−24^% and 5.2 × 10^−25^%, respectively. This result supports the use of the NLME-PBMS method as the only reference in our study, as including the IBMS and SP-PBMS method in the model averaging will not lead to a relevant change.

The NLME-PBMS (MA) considers all functions that passed the goodness of fit test with their corresponding Akaike weights. The relative deviation RDs and the RMSEs were calculated as follows:

(21) $${\mathrm{RD}}_{j}=\frac{{\mathrm{TIA}}_{j}-{\mathrm{TIA}}_{\mathrm{MA}}}{{\mathrm{TIAC}}_{\mathrm{MA}}}$$

(22) $${\mathrm{RMSE}}_{j}=\sqrt{{\left({\mathrm{SDRD}}_{j}\right)}^{2}+{\left({\mathrm{MeanRD}}_{j}\right)}^{2}}$$

where RD is the relative deviation, ${\mathrm{TIA}}_{j}$ is the TIA value calculated using method *j*, and ${\mathrm{TIA}}_{\mathrm{MA}}$ is the TIA calculated using the MA method. ${\mathrm{RMSE}}_{j}$ is the root-mean-square error over all patients of ${\mathrm{RD}}_{j}$, ${\mathrm{SDRD}}_{j}$ is the standard deviation of ${\mathrm{RD}}_{j}$, ${\mathrm{MeanRD}}_{j}$ is the mean of ${\mathrm{RD}}_{j}$ and j corresponds to the IBMS or SP-PBMS or NLME-PBMS methods.

## 3 Results

The fittings using the NLME method for functions $f_{4b}$, $f_{5a}$, $f_{5b}$, $f_{5c}$, $f_{6a}$ and $f_{6b}$ did not pass the goodness-of-fit test, i.e. the fitting failed based on the visual inspection of the fitted curves and/or the CV > 0.50 of the fitted fixed effects (Table 1). Function $f_{3a}$ was selected as the function most supported by the data in the NLME-PBMS approach based on the Akaike weight of 51.72 % (Table 1). The Jackknife method was applied to the subset functions $f_{3a}$, $f_{3c}$ and $f_{4a}$ as these functions had the highest Akaike weights (Table 1). Based on the Jackknife method, the selection of $f_{3a}$ was relatively stable with a median Akaike weight of 50% and range of 38%–74% (Table 1). Function $f_{3a}$ had the highest Akaike weight in 12/13 Jackknife simulations. Therefore, in one case $f_{3c}$ had a higher Akaike weight than $f_{3a}$ (Table 1).

**Table 1:** Goodness of fits and Akaike weights for the NLME model with different SOE functions. The total number of biokinetic data *N* used in this analysis is 46; the number of parameters of the NLME model for the corresponding SOE function is given in column *K*.

Equation number	Function name	K	Coefficient of Variation CV (max)Ϯ	Akaike weight (%)‡	Jackknife Akaike weights (% mean (SD); % median [min, max])§
1	$f_{2a}$	5	0.07	3.11	–
2	$f_{3a}$	7	0.20	51.72	54 (11); 50 [38, 74]
3	$f_{3b}$	7	0.16	3.84	–
4	$f_{3c}$	7	0.21	22.37	33 (10); 33 [19, 59]
5	$f_{4a}$	9	0.27	18.96	13 (7); 14 [2,22]
6	$f_{4b}$	9	0.78	–	–
7	$f_{5a}$	11	Infinity	–	–
8	$f_{5b}$	11	Infinity	–	–
9	$f_{5c}$	11	1.26	–	–
10	$f_{6a}$	13	1.62E+12	–	–
11	$f_{6b}$	13	0.91	–	–

Ϯ The maximum value of the CVs of the fit parameters (CV is calculated as $\sqrt{\exp\left(\omega^{2}\right)-1}$[19], with $\omega^{2}$ being the variance of the fixed effect). Only CV values <0.50 pass the goodness-of-fit check (Table 1 in [7]).

‡ Models passing the goodness-of-fit test are included in the NLME-PBMS (MA) model.

§ The Jackknife analysis was based on functions $f_{3a}$, $f_{3c}$ and $f_{4a}$.

**Figure 1:**
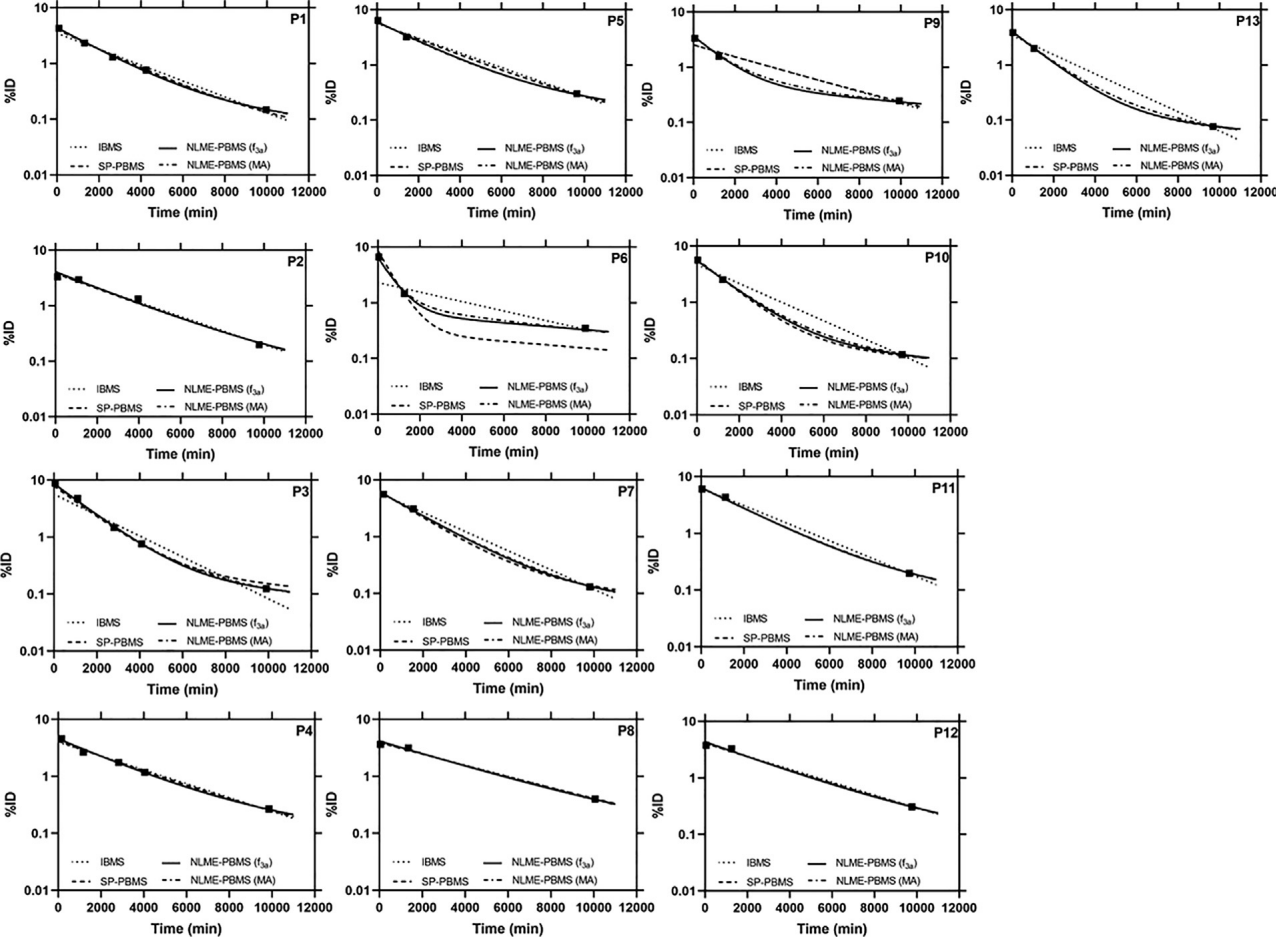
Time-activity data and fit curves were obtained from the IBMS, SP-PBMS, NLME-PBMS ($f_{3a})$ and NLME-PBMS (MA) methods.

**Figure 2:**
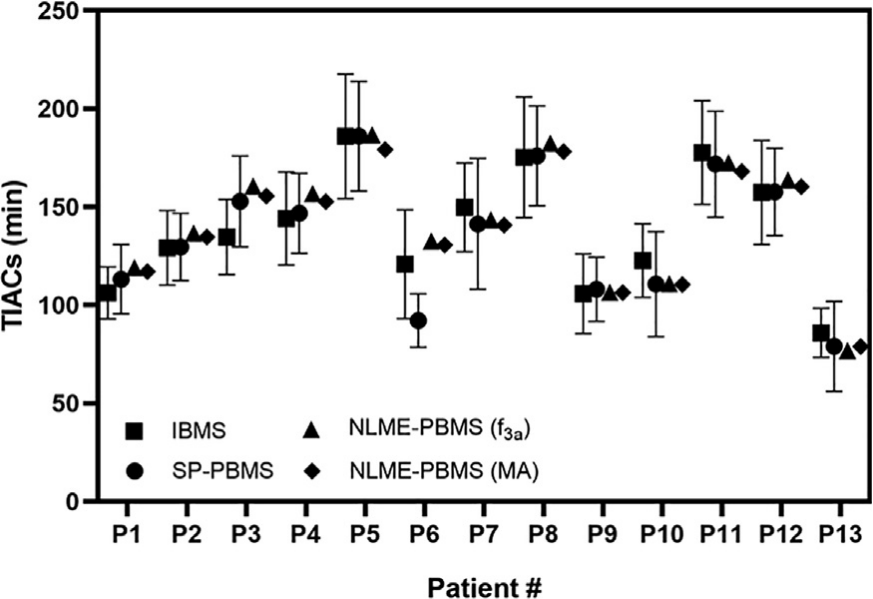
Time-integrated activity coefficients (TIACs) calculated using the NLME-PBMS ($f_{3a}$), NLME-PBMS (MA), IBMS ($f_{2a})$ and SP-PBMS ($\overset{¯}{f_{3b}}\;$) methods. The standard deviations of the TIACs in the IBMS and PBMS methods were calculated based on the sampling according to Eq. (24).

**Figure 3:**
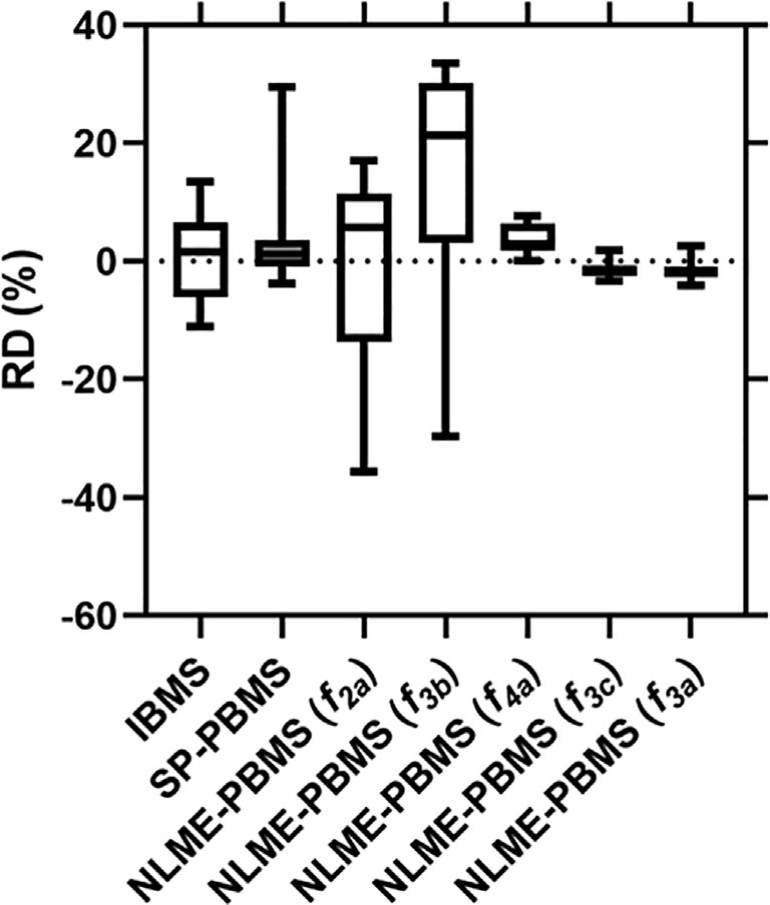
RDs of TIAs obtained from the IBMS, SP-PBMS ($\overset{¯}{f_{3b}}$), NLME-PBMS ($f_{3a}$), NLME-PBMS ($f_{4a}$), NLME-PBMS ($f_{2a}$), NLME-PBMS ($f_{3b}$), and NLME-PBMS ($f_{3c}$) methods.

The five SOE functions, i.e. $f_{3a}$, $f_{3c}$, $f_{4a}$, $f_{3b}$, and $f_{2a}$, were used for the calculation of the TIAs of the NLME-PBMS (MA) method using Eq. (20). Fig. 1 compares the curves obtained from the IBMS ($f_{2a}$), SP-PBMS ($\overset{¯}{f_{3b}}\;$), NLME-PBMS ($f_{3a}$), and NLME-PBMS (MA) methods. The reference model NLME-PBMS (MA) shows a good performance in determining the biokinetic data in all patients (Fig. 1). Visual inspection of the fitted graphs in Fig. 1 shows that the NLME-PBMS ($f_{3a}$) method has a better or equivalent performance as the SP-PBMS and IBMS methods and is similar to the NLME-PBMS (MA) method. Fig. 2 shows the comparison of the TIAs calculated using the IBMS, SP-PBMS, NLME-PBMS ($f_{3a}$) and NLME-PBMS (MA) methods. The SDs of the TIAs per-injected activity or time-integrated activity coefficient (TIACs) in Fig. 2 were calculated based on the bootstrap method with sampling according to Eqs. (17), (18), (19). In general, the TIACs from all methods were similar except for P6. Fig. 3 and Table 2 show the RD of the TIAs of the IBMS, SP-PBMS ($\overset{¯}{f_{3b}}\;$), NLME-PBMS ($f_{3a}$), NLME-PBMS ($f_{4a}$), NLME-PBMS ($f_{2a}$), NLME-PBMS ($f_{3b}$), and NLME-PBMS ($f_{3c}$) methods to the TIAs calculated using NLME-PBMS (MA) method. The NLME-PBMS ($f_{3a}$) has a better performance than the IBMS and SP-PBMS ($\overset{¯}{f_{3b}}\;$) by a factor of four based on the RMSEs values (Table 2).

**Table 2:** The RDs and RMSEs of different methods in comparison to the NLME-PBMS (MA) reference.

Methods	RD (%)		RMSE of the RD (%)
	Mean (SD)	Median [min, max]	
IBMS	1 (7)	2 [−11, 13]	7.4
SP-PBMS	3 (8)	1 [−4, 29]	8.8
NLME-PBMS ($f_{2a}$)	−2 (16)	6 [−36, 17]	16.5
NLME-PBMS ($f_{3a}$)	−2 (2)	−2 [−4, 3]	2.4
NLME-PBMS ($f_{3b}$)	14(19)	21 [−30, 34]	23.5
NLME-PBMS ($f_{3c}$)	−1 (1)	−2 [−3, 2]	2.0
NLME-PBMS ($f_{4a}$)	4 (3)	3 [0, 8]	4.5

## 4 Discussion

Accurate determination of individual TIAs is highly desirable in MRT. Recently, it has been shown that the NLME method could be used for dosimetry in MRT with a relatively accurate determination of TIAs [11], [12]. The advantages of the NLME model are the ability of the method to describe the kinetics of radiopharmaceutical drugs in terms of intra- and inter-individual variability [12], [19]. The information on intra- and inter-individual variability may benefit specific applications such as a single-time-point dosimetry approach [11], [12] in MRT. However, model selection is needed to determine the best fit function with the NLME model approach. Model selection has been shown as an essential and critical aspect of scientific data analysis [8]. Using a model selection procedure for NLME model fitting could increase the reproducibility of the fitting results in contrast to applying the rule of thumbs [24] or simply guessing by the user. The selection of a suitable mathematical model (i.e. function) for calculating TIAs is essential, as using an improper function might invalidate or deteriorate the result [6]. The strong influence of the chosen function on the calculation of RDs can be seen in our analysis results (Fig. 3 and Table 2). Therefore, in this study, we applied the NLME model with model selection to calculate the TIAs. The output of the NLME-PBMS method was then compared with the IBMS and SP-PBMS methods to check if the NLME-PBMS method could be used to determine accurate TIAs in MRT.

Although the functions $f_{3a}$ and $f_{3c}$ are equivalent functions differing only by their parameterisation, function $f_{3a}$ was selected as the best model based on the Akaike weight. This result demonstrates the relevance of model selection as even equivalent functions may lead to a different performance in determining the biokinetic data of radiopharmaceuticals (Fig. 3 and Table 2) due to the assumed difference in the distribution of parameters.

P6 in Fig. 1 demonstrates how the chosen fit function may lead to a considerable difference in TIAs: The fitted curves from the NLME-PBMS ($f_{3a})$ method seem to be more adequate than the IBMS and SP-PBMS methods in describing the biokinetic data in P6. Accordingly, the NLME-PBMS ($f_{3a})$ RMSE value is around four times lower than that of IBMS and SP-PBMS (Table 2). However, one disadvantage of the NLME model approach is that the standard deviation of individual TIAs from the NLME-PBMS method is not analysed as the output of the NLME model only reports the intra-individual variability in the population.

The best model selected from the NLME-PBMS method, i.e. $f_{3a}$, has a similar structure to the best model selected from the SP-PBMS method, i.e. $\overset{¯}{f_{3b}}\;$ (i.e. $f_{3b}$, Eq. (3) with α = 0.9632) [6]. Calculating the TIAs using $\overset{¯}{f_{3b}}\;$ (SP-PBMS, which has 2 adjustable parameters) [6] needs at least three data to obey the constraint of K_max_ (maximum number of fitted parameters) = N (number of data) + 1 of the individual fitting of a new patient. In contrast, the fitting of the NLME model uses a population-based fit, where the ratio between the number of data and the number of fitted parameters is higher than in the individual fits [12]. This allows its use in single-time-point dosimetry.

A general problem in MRT is that it is unknown which function fits the available data best. This applies to the case with many data and with few data per organ. Therefore, a different modeller might use other functions for the same dataset, leading to reproducibility issues [6]. Here the proposed method will provide a more reproducible approach as the model selection includes many suitable SOE functions. Available population data in nuclear medicine are usually heterogeneous and sparse. The presented method can be used for this common situation, as pharmacokinetic information of heterogeneous data can be derived from a population and introduced for the individual fit. Selecting the function most supported by the data [9], [10] and using it in the NLME model fitting is very useful also for other applications in molecular radiotherapies, such as in single-time-point imaging dosimetry [11], [12]. Presumably, when a better fit function is used, the accuracy of single-time-point dosimetry also becomes higher.

In the clinical setting, the presented model selection method would be essential in the sequence of steps as follows: (1) collect biokinetic data of a patient population, (2) derive the fit function most supported by the data as presented here using the NLME-PBMS method, and (3) perform dosimetry for new patients using the derived best function and the NLME model fitting with the known inter- and intra-individual variabilities. The fitted parameters from the previously measured population can be used as the starting values for the fitting of the new patients. Another possibility to decrease the total number of the adjustable parameters is by fixing the parameters of the population to the values from the previous population.

In this study, a maximum of five data per patient were available. More data per patient could yield a different best function (with more parameters) for the NLME-PBMS method, even in the same patient population for the same organ and radiopharmaceutical. Therefore, the NLME-PBMS approach needs to have enough data per patient for the patient population used to determine the best fit function. If the aim is the accurate determination of the TIAs, “enough” data can be determined based on the change in the determined TIAs when increasing the number of data per patient (and possibly also the number of parameters of the best fit function).

Compartmental models, including additional physiological information in the investigated function set, could further improve the NLME-PBMS method results. Compartmental models might perform better than the investigated fit functions based on sum of exponential functions, which are most frequently used for curve fitting in dosimetry in molecular radiotherapy.

In this study, function $f_{3c}$ seems to be better than $f_{3a}$ based on the RMSE values (Table 2). This is in contrast to the Akaike weight results which show $f_{3a}$ as the best function obtained from the NLME-PBMS method (Table 1). This reflects that Akaike weight and RMSE values are different measures. The AICc is used to find the model most supported by the data; however, this tells nothing about the difference in the TIAs for the different functions. Therefore, the differences in TIAs were quantified using the RMSE. In addition, both have an associated uncertainty. The differences between the two measures noted here may disappear if more data and patients are used. Another model selection procedure, the Bayesian Information Criterion (BIC) [25], was also used for our data. As a result, the BIC favours the same best function as the Akaike weight.

The method presented in this study is not constrained to the function set used in this manuscript. Thus, “arbitrary” functions, which should be suited to the kinetics [8], can be added to the here investigated function set, and the best fit function can be determined according to the presented algorithm. Therefore, another function could be the best for a different biokinetic data set (e.g. more patient data at different time points, or for different organs and radiopharmaceuticals).

The advantages of the proposed method are achieved by improving both inputs, i.e. (1) the data and (2) the set of models from which the best one is selected. This, in turn, also improves the results for the TIAs:

1. Data of a population used in the NLME-PBMS model instead of just a single patient in the IBMS method for estimating TIAs is advantageous. Fitting three biokinetic data using IBMS only allows a mono-exponential function with a maximum of two parameters as the degree of freedom cannot be zero (degree of freedom = N–K [6]). We show that the mono-exponential function could lead to inaccurate determination of TIAs in P6 (e.g. Figure 1, Figure 3). By using the NLME-PBMS model, we could fit an SOE function with a higher number of parameters to patients with a low number biokinetic data.
2. The set of models from which the best one is selected using the Akaike weights is also constrained by *K_max_ = (N* − *2)* (Eq. (21)) [7], [8], [9], [26] in both the IBMS and the PBMS methods. Therefore, in our example, the NLME model, in principle, would allow including a much higher number of parameters in the model set functions. However, as seen in Table 1, SOE functions with more than four parameters are unnecessary (based on this biokinetic data set).

The limitations of this study are:

1. Quantification of the activities was based on planar images. It has been shown that activity quantification using imaging with three-dimensional approaches such as SPECT and PET is superior to planar imaging [27]. Planar imaging suffers from biases, and the background correction is known to be sensitive to region-of-interest definition [27], [28]. It is recommended that biokinetic data from three-dimensional approaches be used for future studies. As the main aim of this study was to introduce and present an NLME-PBMS method, which can be implemented for any quantitative imaging data, the results obtained with planar imaging from this study should be translatable to the case of SPECT and PET imaging. Improved quantification should, however, improve the population approach. Reducing systematic and random quantification errors will probably result in changed fixed effects, reduced random effects (inter-individual variability), and reduced intra-individual variability.
2. Quantification without scatter correction is a limitation in our study. The presented population method is constructed to use similarities in the biokinetics of the population under investigation. Thus, patients must be measured similarly for the method to be applicable. Therefore, removing scatter from the data by implementing quantitative imaging is expected to improve the results obtained using this population-based method, as discussed in item 1 above.
3. The starting values of the adjustable parameters were chosen based on a trial-and-error approach. This could lead the minimisation algorithm to local minima of the objective function. Therefore, different starting values were investigated. Of these, only fit results showing a good fit based on the goodness of fit criteria having the highest likelihood were used for further evaluation.

## 5 Conclusions

A critical unresolved issue in MRT dosimetry is the question of which fit function to use for calculating the time-integrated activity. We developed a method to include fitting function selection in a population-based method to determine the best fit function for calculating TIAs in MRT for a given radiopharmaceutical, organ and patient data set. The best fit function can then be used to fit a “new” patient with the known inter- and intra-individual variabilities from the corresponding patient population. The technique combines standard practice approaches in pharmacokinetics, i.e. an Akaike-weight-based model selection and the NLME model framework. The fit function selection accuracy and precision in the NLME-PBMS method are less dependent on noise in the data than in the IBMS method, which relies on only very few data of a single patient. Thus, using the proposed fit function selection and the NLME model will increase the reproducibility of the found best fit function for determining TIAs.

## Consent for Publication

All authors read the manuscript and consented to its publication.

## CRediT authorship contribution statement

**Deni Hardiansyah:** Conceptualization, Methodology, Software, Validation. **Ade Riana:** Software, Validation. **Matthias Eiber:** Resources. **Ambros J. Beer:** Resources. **Gerhard Glatting:** Conceptualization, Methodology, Validation, Resources.

## Declaration of Competing Interest

The authors declare that they have no known competing financial interests or personal relationships that could have appeared to influence the work reported in this paper.
